# Antibacterial, antifungal, and phytochemical properties of *Salsola kali* ethanolic extract

**DOI:** 10.1515/biol-2022-0962

**Published:** 2024-09-03

**Authors:** Shimaa Bashir, Said Behiry, Abdulaziz A. Al-Askar, Przemysław Łukasz Kowalczewski, Haitham H. Emaish, Ahmed Abdelkhalek

**Affiliations:** Plant Protection and Biomolecular Diagnosis Department, Arid Lands Cultivation Research Institute, City of Scientific Research and Technological Applications, Alexandria, 21934, Egypt; Agricultural Botany Department, Faculty of Agriculture (Saba Basha), Alexandria University, Alexandria, 21531, Egypt; Department of Botany and Microbiology, College of Science, King Saud University, P.O. Box 2455, Riyadh, 11451, Saudi Arabia; Department of Food Technology of Plant Origin, Poznań University of Life Sciences, Poznań, Poland; Department of Soils and Agricultural Chemistry, Biosystem Engineering, Faculty of Agriculture (Saba Basha), Alexandria University, Alexandria, 21531, Egypt

**Keywords:** *Salsola kali*, antifungal activity, antibacterial activity, phytochemical, HPLC, GC-MS

## Abstract

The research into the use of plants as plentiful reservoirs of bioactive chemicals shows significant potential for agricultural uses. This study focused on analyzing the chemical composition and potency of an ethanolic extract obtained from the aerial parts (leaves and stems) of *Salsola kali* against potato pathogenic fungal and bacterial pathogens. The isolated fungal isolates were unequivocally identified as *Fusarium oxysporum* and *Rhizoctonia solani* based on morphological characteristics and internal transcribed spacer genetic sequencing data. The antifungal activity of the extract revealed good inhibition efficacy against *R. solani* (60.4%) and weak activity against *F. oxysporum* (11.1%) at a concentration of 5,000 µg/mL. The *S. kali* extract exhibited strong antibacterial activity, as evidenced by the significant inhibition zone diameter (mm) observed in all three strains of bacteria that were tested: *Pectobacterium carotovorum* (13.33), *Pectobacterium atrosepticum* (9.00), and *Ralstonia solanacearum* (9.33), at a concentration of 10,000 µg/mL. High-performance liquid chromatography analysis revealed the presence of several polyphenolic compounds (μg/g), with gallic acid (2942.8), caffeic acid (2110.2), cinnamic acid (1943.1), and chlorogenic acid (858.4) being the predominant ones. Quercetin and hesperetin were the predominant flavonoid components, with concentrations of 1110.3 and 1059.3 μg/g, respectively. Gas chromatography-mass spectrometry analysis revealed the presence of many bioactive compounds, such as saturated and unsaturated fatty acids, diterpenes, and phytosterols. The most abundant compound detected was *n*-hexadecanoic acid, which accounted for 28.1%. The results emphasize the potential of *S. kali* extract as a valuable source of bioactive substances that possess good antifungal and antibacterial effects, which highlights its potential for many agricultural uses.

## 1 Introduction

Plants are continually exposed to a wide range of pests and microbial pathogens in their environment. Plant diseases significantly reduce crop quality and yield or limit production, causing severe agricultural losses [1,2]. In tropical and subtropical regions, fungi are the primary cause of many plant diseases, which leads to a substantial reduction in agricultural output [3]. Pathogenic fungi may cause serious diseases in a wide variety of flowering plants. Some fungal species have a wide host range, while others are extremely host-specific [4]. Based on their relationship with the host, fungal pathogens are divided into three categories: biotrophic, which feeds on living cells; necrotrophic, which kills and feeds on dead cells; and hemibiotrophic, which follows a biphasic strategy [5]. There is evidence that necrotrophic fungi are more economically harmful than biotrophic ones. Both *Rhizoctonia solani* and *Botrytis cinerea* are typical examples of necrotrophic pathogens that infect a wide range of dicot plants, and they are linked closely to symptoms of gray mold and root or rot disease [6,7,8]. On the contrary, *Fusarium oxysporum* is a host-specific hemibiotrophic pathogen that infects plant vascular tissue, causing *Fusarium* wilt disease [9].

Although bacterial diseases of plants are less common than fungal ones, the economic loss caused by these diseases is massive [10]. Bacterial plant pathogens are worldwide in disruption and have wide host ranges. The majority of agriculturally important crops, such as potatoes, tomatoes, eggplants, peppers, etc., are susceptible to a variety of bacterial infections [11]. There are over 150 bacterial species that cause various plant diseases, and they have been divided into the following three families: Xantomonadaceae, Pseudomonaceae, and Enterobacteriaceae [12]. *Dickeya*, *Pectobacterium*, *Ralstonia,* and *Xanthomonas* are the most common genera that cause blackleg, bacterial soft rot, brown rot, and spot diseases, respectively [13,14]. Pathogenic bacteria penetrate plant tissue and cause cell death by secreting lytic enzymes that degrade and destroy cellular structure. In addition to these enzymes, bacteria also produce exopolysaccharides, toxins, and phytohormones, which are all virulence factors that help them cause disease [15].

Chemical pesticides are the primary method for controlling plant diseases [16]. The continued use of pesticides has a negative influence on the surrounding environment and public health. These harmful effects are a result of the high toxicity and lack of biodegradability of pesticides [17,18]. Therefore, using natural materials as a form of disease management is a novel approach to agricultural development. Biopesticides are a term that refers to pesticides made from natural sources. In comparison to synthetic pesticides, biopesticides have a shorter half-life in the environment, thus reducing possible environmental effects and delaying pathogen resistance development [19].

The *Salsola* genus, from the Amaranthaceae family (previously Chenopodiaceae), is an annual and perennial herb, shrub, and semi-shrub plant that grows in places with low annual rainfall and saline soils [20]. The genus names originate from the Latin words “salsus” or “sallere,” which means “to salt.” This genus, which consists of about 100–150 species, is common along seashores, in grasslands, and in semiarid and arid regions. *Salsola* species are resistant to pH, heat, water, and salt stresses, and they represent approximately 45% of desert plants [21]. Several plants belonging to the genus *Salsola* are used in the pharmaceutical and cosmetic industries to treat cough, influenza, and skin diseases, as well as traditional medicine. The aqueous ethanolic extract of *Salsola cyclophylla* exhibited the most significant analgesic effect, reducing pain by 89.86, 87.50, and 99.66% after 60, 90, and 120 min, respectively. Additionally, this extract showed the highest anti-inflammatory activity, with reductions of 41.07, 34.51, and 24.82% after 2, 3, and 6 h of administration, respectively. Because of its relatively high crude protein content, the plant is also utilized as animal feed during times of difficulty and drought [22,23]. Additionally, some species of the genus *Salsola* have been described as having industrial benefits. For example, *Salsola soda* and *Salsola kali* were used to make sodium carbonate, linin, cotton bleaching, glass, and soap [20,24]. The *Salsola* genus contains an abundance of different types of phytoconstituents, the majority of which are flavonoids, phenolic compounds, nitrogenous compounds, saponins, triterpenes, sterols, fatty acids, and volatile constituents [21,25,26]. Plants in the genus *Salsola* have antimicrobial and antioxidant effects that are generally attributed to secondary metabolic products. The plant extracts of *Salsola vermiculata* and *Salsola cyclophylla*, which contain a high concentration of flavonoids, steroids, and alkaloids, showed significant antimicrobial activity against *Staphylococcus aureus*, *Klebsiella pneumonia*, and *Candida albicans* [27,28]. Oueslati et al. [26] stated that the chloroform fractions of *Salsola villosa* displayed promising activity against Gram-positive and Gram-negative bacteria. Recently, nine organic compounds were detected as major constituents of the aerial part of *S. kali*, which is shown to have antimicrobial activity against *S. aureus*, *Staphylococcus epidermidis*, and *Candida kefyr* [29]. The utilization of extracts derived from *Salsola* plants as biocontrol agents for plant diseases is now in its nascent phase. This investigation aims to analyze the phytochemical composition of *Salsola kali* extract utilizing high-performance liquid chromatography (HPLC) and gas chromatography-mass spectrometry (GC-MS) techniques, with the objective of assessing its potential efficacy against three bacterial and two fungal plant pathogens.

## 2 Materials and methods

### 2.1 Collection and plant identification

The *S. kali* plant was collected from Borg El-Arab City, Alexandria, Egypt, at coordinates 30°54′25.9ʺN 29°32′41.3ʺE. The plant material was taxonomically identified at the Plant Production Department, Faculty of Agriculture (Saba Basha), Alexandria University, Egypt. We also performed a molecular identification to confirm the plant taxonomy’s identity. Fresh plant tissue was collected, and genomic DNA was extracted using the cetyltrimethylammonium-bromide method [30]. The tissue was ground to a fine powder in liquid nitrogen, and an extraction buffer was added to facilitate DNA extraction. Following extraction, DNA purification was performed using phenol/chloroform/isoamyl alcohol, and the DNA was precipitated with isopropanol. The DNA pellet was washed with ethanol, air-dried, and resuspended in tris-ethylenediaminetetraacetic acid (EDTA) buffer (10 mM Tris–HCl, 1 mM EDTA, pH 8.0). DNA quality and quantity were assessed using agarose gel electrophoresis and a spectrophotometer. The internal transcribed spacer (ITS) region of nuclear ribosomal DNA was amplified using the primers ITS1 (5′-TCCGTAGGTGAACCTGCGG-3′) and ITS4 (5′-TCCTCCGCTTATTGATATGC-3′). A polymerase chain reaction (PCR) mix was prepared containing genomic DNA, 10× Taq buffer, 25 mM MgCl_2_, 2.5 mM dNTP mix, 10 µM of each primer, and Taq DNA polymerase. The PCR conditions included an initial denaturation at 95°C for 2 min, followed by 35 cycles of denaturation at 95°C for 1 min, annealing at 55°C for 45 s, and extension at 72°C for 1 min. The amplification was completed with a final extension step at 72°C for 5 min. The PCR product size was confirmed by running it on a 1% agarose gel stained with redsafe stain. For the ribulose-1,5-bisphosphate carboxylase/oxygenase large subunit (rbcL) gene, the primers rbcL1 (5′-ATGTCACCACAAACAGAAAC-3′) and rbcL724R (5′-TCGCATGTACCTGCAGTAGC-3′) were used. A PCR reaction mix similar to that used for the ITS region was prepared. The thermal cycling conditions were: initial denaturation at 94°C for 3 min, followed by 35 cycles of denaturation at 94°C for 30 s, annealing at 55°C for 30 s, and extension at 72°C for 1 min. A final extension at 72°C for 10 min was performed. Gel electrophoresis was used to validate amplification. Purification of the PCR products was carried out using a PCR purification kit from QIAquick (Cat. No.28104, Hilden, Germany). The purified products were sequenced using the Sanger sequencing method, with the same primers used for PCR amplification. The sequencing results were analyzed using bioinformatic tools. BLAST was used to find matches between sequences and GenBank reference databases in order to identify plant species based on the ITS region and rbcL gene sequences.

### 2.2 Crude extract preparation

Before the extraction process, the aerial parts of the plant (leaves and stems) were washed with water to remove soil particles and air-dried at room temperature. The dried plant materials were then powdered using a blender and sieved through a 100-mesh stainless steel sieve. The maceration procedure extracted approximately 50 g of powdered aerial portions of the *S. kali* plant with 150 mL of absolute ethanol as a solvent [31]. The combination was allowed to soak for 3 days under laboratory conditions and darkness. To eliminate any plant debris, the extract was filtered through Whatman filter paper No. 1, and the solvent was evaporated with a rotary evaporator to yield the *S. kali* extract. The crude extract was generated in different concentrations and emulsified using dimethyl sulfoxide (DMSO).

### 2.3 Source of the phytopathogenic microorganisms and their identification

Bacterial strains used in this study are listed in Table 1. Meanwhile, the fungal isolates were isolated from potato plants and tubers that showed dry rot or black scurf. The infected potato tissues were washed with tap water to eliminate soil particles. We subjected decaying tuber fragments to a 2% sodium hypochlorite solution for a duration of 5 min. Subsequently, we rinsed the samples three times with sterile distilled water, each rinse lasting 5 min. Finally, we dried the samples using sterile filter paper. About 0.5-cm fragments that had been disinfected were placed on potato dextrose agar (PDA) media. Each plate contained four pieces, which were then cultivated at a temperature of 25°C for 5–7 days. Multiple fungal colonies were identified on the PDA medium; however, only colonies resembling *Fusarium* or *Rhizoctonia*-like were selected. For *Fusarium*, the fungal isolate was initially identified based on its physical characteristics using the morphological criteria outlined by Leslie and Summerell [32]. Following the steps outlined by Stefańczyk and Sobkowiak [33], the subcultures that came from the outermost part of the colony were separated by letting the conidia germinate on water agar at 16°C for 1–2 days. *Rhizoctonia*-like colonies were identified based on their morphological characteristics, as outlined by Rliizoctonja and Solan [34]. The ITS amplification region was used to confirm the species molecular characterization of the fungal isolates that were collected [35]. These were then sequenced at Macrogen Company in Seoul, Korea. The sequences were submitted to the GenBank website and aligned with the most related strains. To obtain the accession numbers, the identified isolates were deposited at the NCBI.

**Table 1:** List of phytopathogenic microorganisms used in this study

Classification	Strain	Accession number	References
Phytopathogenic fungi	*F. oxysporum*	This study	—
	*R. solani*	This study	
Phytopathogenic bacteria	*Pectobacterium carotovorum*	HF674984	[36]
	*Pectobacterium atrosepticum*	MG706146	
	*Ralstonia solanacearum*	GH425351	

### 2.4 Antifungal assay of the plant extract

The mycelia growth rate method was employed to investigate the antifungal efficacy of *S. kali* extract against three phytopathogenic fungi [37]. The fungal strains were grown on PDA medium for 7 days at 25°C. After this, 5 mm plugs of fungus cut from the edge of the fungus colony were put in the middle of the PDA plates, which have a gradient of plant extract concentrations from 1,000 to 5,000 µg/mL. There are three replicates for each treatment. One set of the plates was used as negative controls, in which DMSO was used instead of the plant extract, while the fungicide carbendazim 50 WP (250 µg/mL) was used in another set as a positive control. In the dark, the cultures were incubated at 25°C for 7 days. For each replicate, the average of two perpendicular mycelia-growth diameters was used to calculate radial mycelial growth. The following formula was used to convert the radial growth rate to a percentage of inhibition. Growth inhibition (%) = [(*C* − *T*)/*C*] × 100, where *C* is the radial growth in the control (mm) and *T* is the radial growth in the presence of plant extract (mm).

### 2.5 Antibacterial assay of the plant extract

The plant extract was screened for antibacterial activity using the well agar method as described by Balouiri et al. [38]. The bacterial strains were pre-cultured in Luria–Bertani broth overnight in a shaking incubator at 30°C. Then, a 0.5 McFarland standard was used to dilute each strain to a concentration of 10^8^ cells/mL. Using a cotton swab, sterile Luria–Bertani agar plates were streaked uniformly with the standardized bacterial broth culture. Wells of equal size (6 mm in diameter) were made into each plate using a sterile cork borer and filled with 50 µL of 3,000, 5,000, 7,000, and 10,000 µg/mL of *S. kali* extract. The positive and negative controls consisted of vancomycin (30 μg/mL) and DMSO (50 µL/well), respectively. All the plates were kept in the refrigerator for 2 h to allow the extracts to diffuse into the agar and then incubated at 30°C overnight. By measuring the diameters of inhibitory zones (including well diameter) on the agar surface around the wells, the sensitivity of the bacterial strains to the plant extracts was calculated.

### 2.6 HPLC analysis of phenolic and flavonoid compounds of the plant extract

The HPLC profile of phenolics and flavonoids in *S. kali* extract was performed using an Agilent 1260 Infinity HPLC Series system with a quaternary pump. The separation was achieved using a Zorbax Eclipse Plus C18 column, 100 mm in length and 4.6 mm in width. A 20 µL of plant extract was filtered through a 0.2 µm syringe filter and then injected into the system. A gradient of 0.2% (v/v) phosphoric acid in HPLC-grade water (mobile phase A), methanol (mobile phase B), and acetonitrile (mobile phase C) was used to separate the substances. The flow rate was kept at 0.7 ml/min, and the absorbance of detection was 273 nm at 30°C [39]. In order to measure the amount of phenolic and flavonoid compounds, the following standards were used: methyl gallate, caffeic acid, syringic acid, pyrocatechol, rutin, ellagic acid, coumaric acid, vanillin, ferulic acid, naringenin, daidzein, quercetin, cinnamic acid, apigenin, kaempferol, and hesperetin.

### 2.7 GC-MS analysis

The chemical composition of the extract from *S. kali* was analyzed using a GC-MS instrument with an Agilent 7000D instrument from Technologies in Santa Clara, CA, USA. The GC-MS instrument was equipped with a column composed of a combination of 5% diphenyl and 95% dimethylpolysiloxane, as well as an HP-5MS capillary column. The carrier gas, composed of 99.99% helium, was controlled to flow at a rate of 1 mL per minute. The scanning duration for ionization energy was set to 70 eV, with a rate of 0.2 s. The fragment detection ranged from 40 to 600 *m*/*z*. The sample was injected into 1 μL aliquots and split in a 10:1 ratio at 250°C. The starting oven temperatures of the column were set at 50°C with a rate of increase of 3 min. They were then gradually increased by 10°C per minute until reaching 280°C. Finally, the temperatures were raised to 300°C with a rate of growth of 10 min. The phytochemical components that were identified were compared to the authentic chemicals found in the Wiley Registry 8E, Replib, and Mainlib libraries [40].

### 2.8 Statistical analysis

The antimicrobial inhibition % and diameter of the inhibition zone were tested three times, and the average was calculated. The data were then examined statistically with a one-way analysis of variance (ANOVA) in the CoStat program, substantially distinguishing the means at the LSD 0.05 level.

## 3 Results

### 3.1 Identification of *S. kali* plant

**Figure 1:**
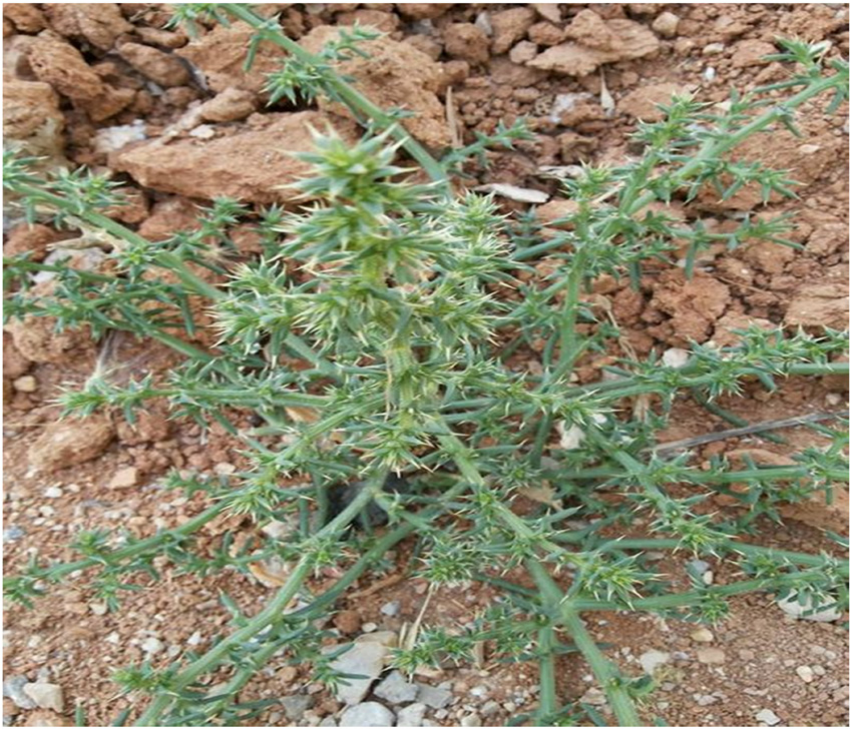
Overview of the *S. kali* whole plant.

The examined plant sample was recognized based on its morphology as *S. kali* (Figure 1) and has been stored in the repository of Alexandria University with the assigned number 7709. The genomic DNA extracted from the plant sample was successfully amplified using PCR for both the ITS region and the rbcL gene. Gel electrophoresis confirmed the presence of PCR products of expected sizes for both markers. Sequencing of the PCR products yielded high-quality sequences for the ITS region and rbcL gene. The sequences were analyzed using bioinformatics tools and compared to reference sequences in GenBank. Based on sequence similarity, the plant sample was identified as *S. kali* and accessioned with numbers PP948524 and PP955420 for ITS region and rbcL, respectively.

### 3.2 *Fusarium* and *Rhizoctonia* isolates identification

**Figure 2:**
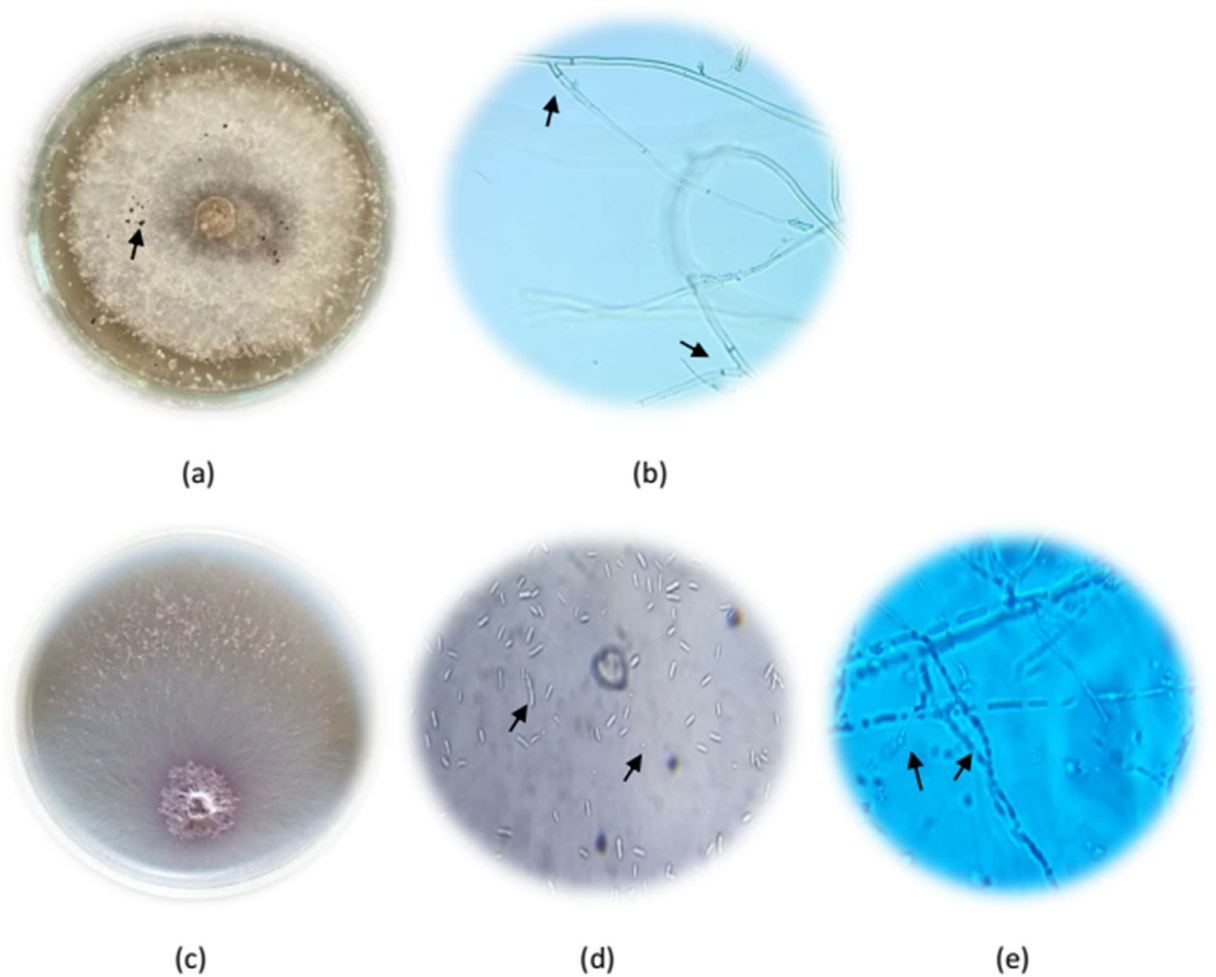
(a) Upper view of hyphal growth of *R. solani* on PDA medium contained black sclerotia. (b) Microscopic features of *R. solani*, showing angle branching of septate hyphae. (c) Upper view hyphal growth of *F. oxysporum* on PDA medium. (d) Microscopic traits of *F. oxysporum*, showing macroconidia and microconidia. (e) Microscopic view of *F. oxysporum*, showing chlamydospores.

The morphological characteristics of the isolated fungi were investigated by observing their growth attributes on PDA and using a light microscope (Figure 2). The isolated *Fusarium* had fast-growing colonies on PDA that were initially white but turned pink to violet with age, presenting a cottony or woolly appearance. Its conidiophores were simple or branched, bearing both monophialides and polyphialides. The macroconidia were fusiform to sickle-shaped, typically 3–5 septate, and slightly curved with pointed ends (Figure 2). The microconidia were ellipsoid to cylindrical, mostly single-celled (Figure 2). All these features correspond to the fungal species *F. oxysporum*. *Rhizoctonia*, in other words, formed light brown to dark brown colonies on PDA with dense, crust-like growth and a smooth, slightly shiny texture. In its hyphae, there were right-angle branching with septa close to the branching point, cells with more than one nucleus, and no clamp connections (Figure 2). All these phenotypic characters indicated that the fungal isolate was *R. solani*. Following this, these isolates were molecularly identified using Sanger sequencing’s ITS region recognition. After alignment in the GenBank database, the fungal isolates were definitively identified as *F. oxysporum* and *R. solani*, and they were deposited under accession numbers OR116509 and OR116529, respectively.

### 3.3 The yield of the *S. kali* extract and its antifungal activity

**Figure 3:**
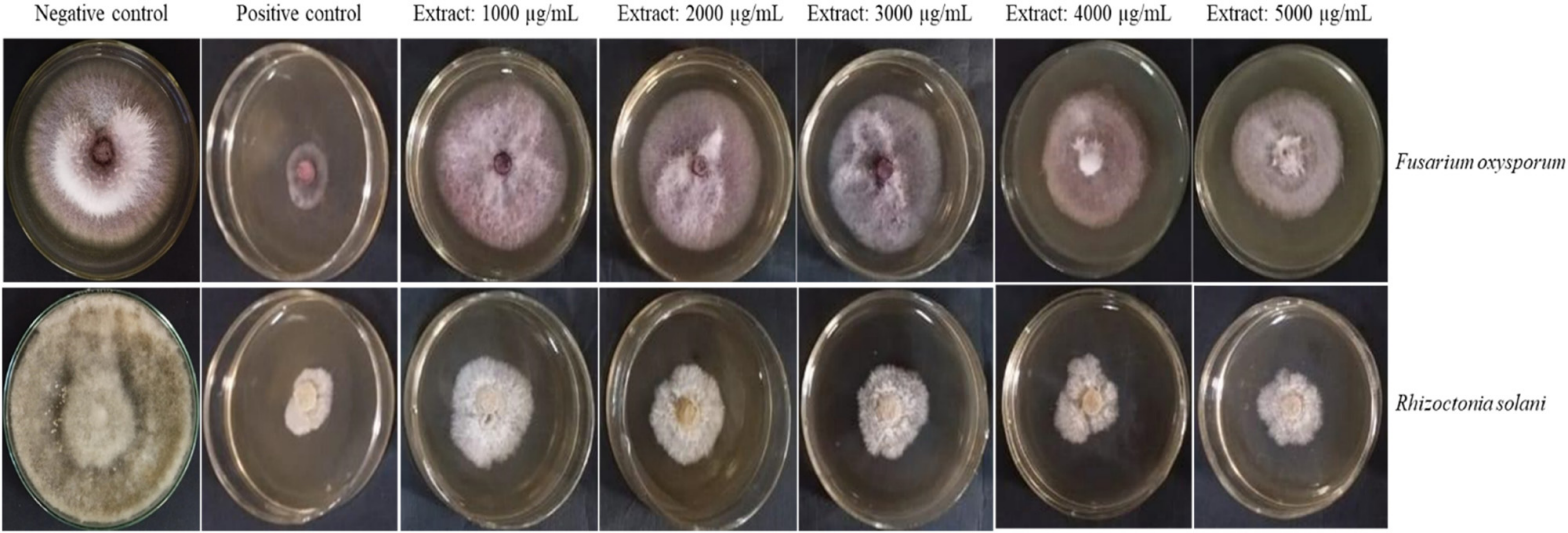
Antifungal activity of the *S. kali* extract against *F. oxysporum* and *R. solani* at 1,000, 2,000, 3,000, 4,000, and 5,000 µg/mL concentrations compared to the positive and negative controls.

The aerial parts of *S. kali* were extracted with ethanol, resulting in 87.4 mg/g of dry matter. The plant extract’s in vitro antifungal activity was assessed by measuring its inhibitory effects on *F. oxysporum* and *R. solani* (Figure 3). Table 2 compares the growth inhibition percentage of fungal strains when exposed to various amounts of the extract with an untreated control for comparison. Out of the fungal strains that were examined, *R. solani* was shown to be the most susceptible to the *S. kali* extract. At a concentration of 5,000 µg/mL, the extract inhibited *R. solani* growth by 60.4%.

**Table 2:** Effect of the *S. kali* extract on fungal growth

Concentration (µg/mL)	Growth inhibition (%)	
	*F. oxysporum*	*R. solani*
1,000	5.6 ± 0.22e	32.9 ± 0.56e
2,000	6.9 ± 0.32d^*^	47.8 ± 0.72d
3,000	8.2 ± 0.42c	55.5 ± 0.39c
4,000	11.1 ± 0.53b	56.2 ± 0.72c
5,000	11.1 ± 0.49b	60.4 ± 0.86b
Negative control	0.0 ± 0.00f	0.0 ± 0.00f
Positive control^**^	53.1 ± 0.89a	89.8 ± 0.97a

*Averages followed by the same letter in each column mean no significant differences. ^**^Carbendazim 50 Wp (250 µg/mL).

### 3.4 Antibacterial activity of *S. kali* extract

**Figure 4:**
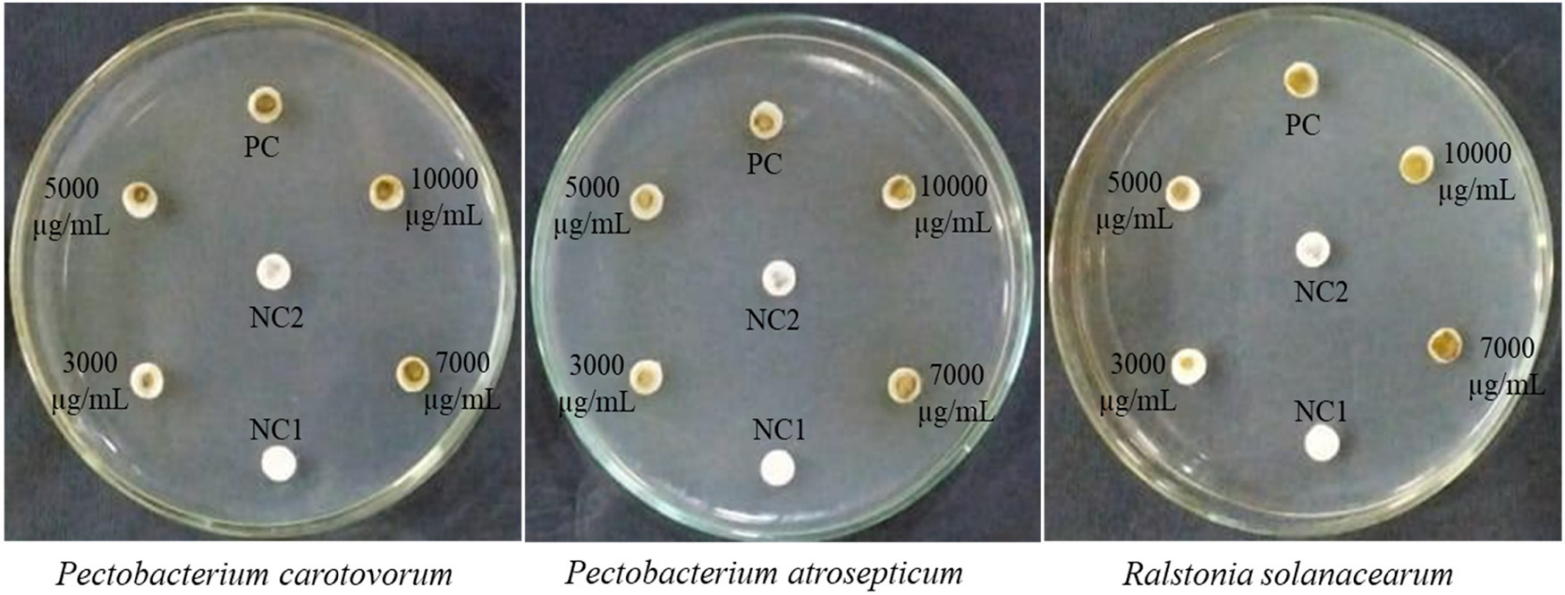
Antibacterial activity of the *S. kali* extract against *P. carotovorum, P. atrosepticum,* and *R. solanacearum* at 3,000, 5,000, 7,000, and 10,000 µg/mL concentrations compared to the positive and two negative controls.

The antibacterial activity of plant extract was assessed by measuring its inhibitory effects on *Pectobacterium carotovorum*, *Pectobacterium atrosepticum,* and *Ralstonia solanacearum* (Figure 4). Table 3 demonstrates that the *S. kali* extract, at different doses, had significant efficacy against all the bacterial strains tested, surpassing the positive control. The plant extract had an inhibitory effect on a range of 6.00–13.33 mm, and at a concentration of 10,000 µg/mL, it had the strongest effect on the bacterial strain *P. carotovorum* (13.33 mm). In general, the findings indicated that there was a statistically significant distinction observed when comparing the impacts of *S. kali* extract at concentrations of 7,000 and 10,000 µg/mL.

**Table 3:** Antibacterial activity of *S. kali* extracts against plant pathogenic bacteria

Concentration (µg/mL)	Inhibition zone diameter (mm) ± standard deviation		
	*P. carotovorum*	*P. atrosepticum*	*R. solanacearum*
3,000	8.33 ± 0.47d^*^	7.67 ± 0.47c	0.00 ± 0.00c
5,000	9.00 ± 0.00cd	8.33 ± 0.47bc	9.00 ± 0.00b
7,000	9.33 ± 0.47c	9.00 ± 0.00b	9.00 ± 0.00b
10,000	13.33 ± 0.47b	9.00 ± 0.00b	9.33 ± 0.47b
Negative control	0.00 ± 0.00e	0.00 ± 0.00d	0.00 ± 0.00c
Positive control^**^	14.33 ± 0.47a	18.67 ± 0.47a	12.67 ± 0.47a

^*^Averages followed by the same letter in each column mean no significant differences. ^**^Vancomycin (30 μg/mL).

### 3.5 HPLC analysis of *S. kali* extract

**Figure 5:**
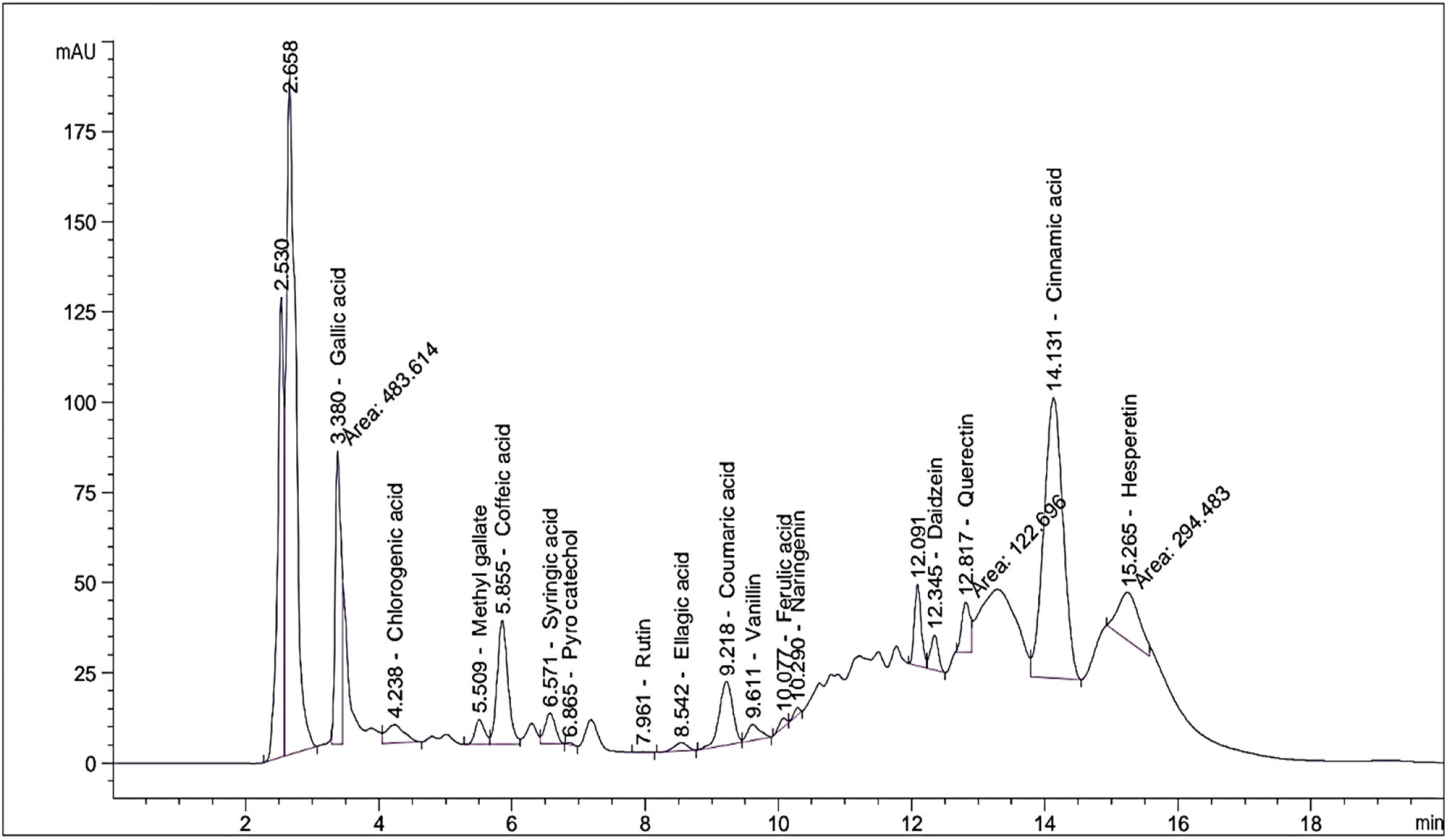
HPLC chromatogram of *S. kali* extract.

An HPLC analysis was conducted on the fractions of *S. kali* to determine the presence of 10 phenolic compounds. These compounds include gallic acid, chlorogenic acid, methyl gallate, caffeic acid, syringic acid, pyrocatechol, ellagic acid, coumaric acid, ferulic acid, and cinnamic acid. According to the data presented in Figure 5 and Table 4, gallic acid had the highest concentration at 2942.8 μg/g, followed by caffeic acid at 2110.2 μg/g, cinnamic acid at 1943.1 μg/g, chlorogenic acid at 858.4 μg/g, and coumaric acid at 517.5 μg/g. Conversely, ferulic acid had a lower concentration at 84.2 μg/g, and pyrocatechol had the lowest concentration at 34.2 μg/g. Table 4 shows the results of our analysis of nine flavonoid compounds in the plant extract. The detected flavonoid compounds were quercetin (1110.3 μg/g), hesperetin (1059.3 μg/g), daidzein (314.3 μg/g), vanillin (163.3 μg/g), naringenin (104.1 μg/g), and rutin (14.1 μg/g). However, catechin, apigenin, and kaempferol were not identified.

**Table 4:** Retention times (RT, min) and concentrations (μg/g) of phenolic and flavonoid compounds present in *S. kali* extract using the HPLC system

Phenolic compounds			Flavonoid compounds		
Compound	RT*	(μg/g)	Compound	RT	(μg/g)
Gallic acid	3.380	2942.845	Catechin	4.549	ND**
Chlorogenic acid	4.238	858.4283	Rutin	7.961	14.1286
Methyl gallate	5.509	266.4428	Vanillin	9.611	163.2905
Caffeic acid	5.855	2110.179	Naringenin	10.290	104.0414
Syringic acid	6.571	435.6735	Daidzein	12.345	314.3267
Pyrocatechol	6.865	34.23431	Quercetin	12.817	1110.315
Ellagic acid	8.542	405.438	Apigenin	14.469	ND
Coumaric acid	9.218	517.4836	Kaempferol	14.948	ND
Ferulic acid	10.077	84.15188	Hesperetin	15.265	1059.257
Cinnamic acid	14.131	1943.147			

*RT = retention time, **ND = not detected.

### 3.6 GC-MS analysis of *S. kali* extract

**Figure 6:**
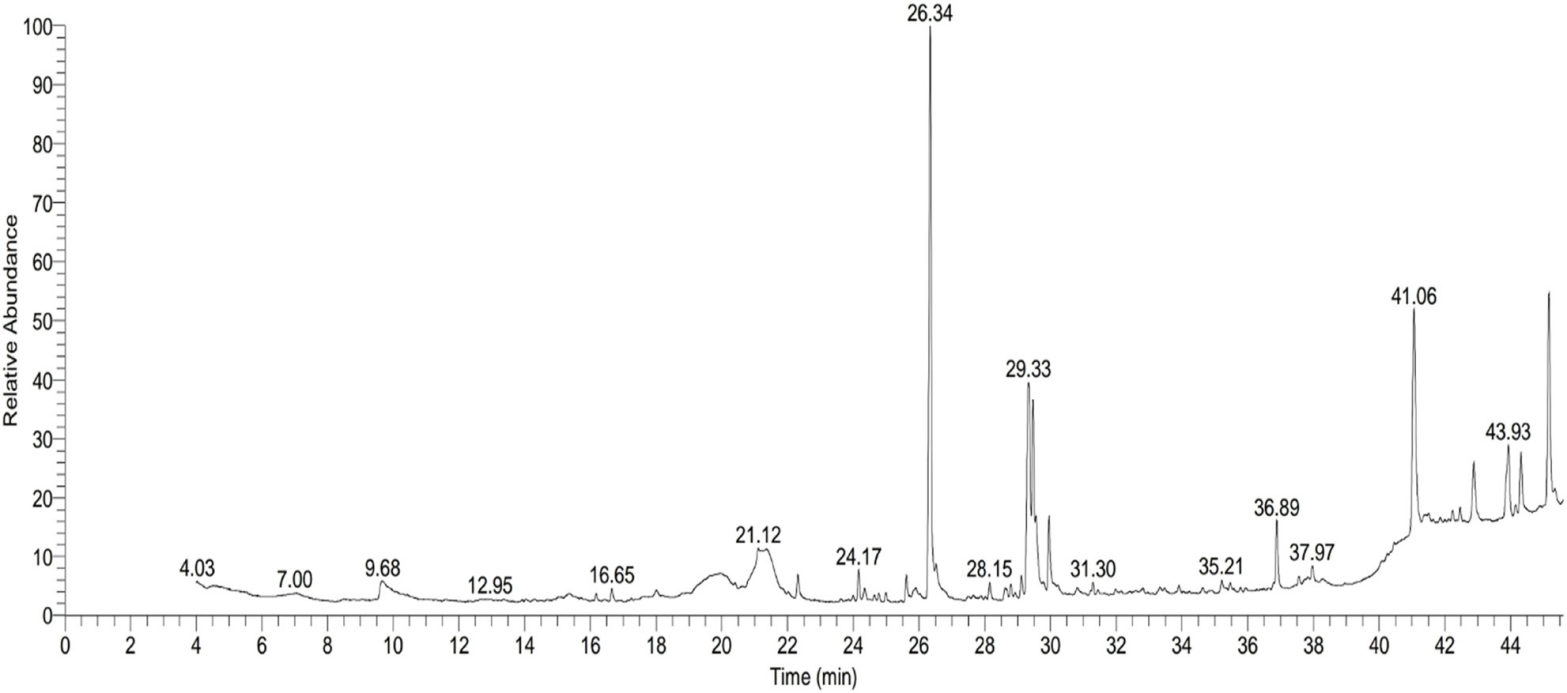
Chromatograms of GC-MS analysis of *S. kali* extract.

GC-MS was used to find the bioactive compounds in *S. kali* extract. The compounds were matched chemically by looking at their retention time, peak areas, molecular weight, and molecular formula against known compounds from various mass spectral libraries. The mass spectra of the phytochemicals identified in *S. kali* extract are shown in Figure 6 and listed in Table 5 in more detail. The identified phytochemicals fell into various classes, including saturated and unsaturated fatty acids, fatty acid esters, dialkyl ethers, diterpenes, triterpenoid derivatives, and phytosterol. Of the 23 compounds identified, the major compound identified was *n*-hexadecanoic acid, which represents 28.03% of the total area, followed by ursolic acid methyl ester (13.22%), 9,12-octadecadienoic acid (12.31%), á-sitosterol (11.14%), and *trans*-13-octadecenoic acid (5.86%).

**Table 5:** Phytochemical constituents identified in the extract of *S. kali* using gas chromatography-mass spectrometry

RT	Area %	Compound	Molecular formula	Compound class
16.65	0.64	Phenol, 2,4-bis(1,1-bimethylethyl)	C_14_H_22_O	Phenol
22.31	1.12	Tetradecanoic acid	C_14_H_28_O_2_	Saturate FA*
24.16	1.31	Ethanol, 2-(9-octadecenyloxy)-, (*Z*)-	C_20_H_40_O_2_	Dialkyl ethers
25.61	1.26	Palmitic acid methyl ester	C_17_H_34_O_2_	FA ester
26.34	28.03	*n*-Hexadecanoic acid	C_16_H_32_O_2_	Saturated FA
26.52	0.54	Vitamin A palmitate	C_36_H_60_O_2_	Retinyl esters
28.15	0.84	Hexadecanoic acid	C_19_H_38_O_4_	Saturated FA
28.79	0.60	Methyl 9,9-dideutero octadecanoate	C_19_H_36_D_2_O_2_	FA
29.11	0.98	Tetraneurin-A-diol	C_15_H_20_O_5_	Sesquiterpene lactone
29.31	12.31	9,12-Octadecadienoic acid	C_18_H_32_O_2_	Unsaturated FA
29.47	5.86	*trans*-13-Octadecenoic acid	C_18_H_34_O_2_	Unsaturated FA
29.56	1.96	*cis*-Vaccenic acid	C_18_H_34_O_2_	Unsaturated FA
29.96	3.49	Octadecanoic acid	C_18_H_36_O_2_	Saturated FA
31.30	0.53	Tributyl acetylcitrate	C_20_H_34_O_8_	citric acid esters
36.88	3.28	Phorbol	C_20_H_28_O_6_	Diterpenes
37.96	0.74	4-Hexyl-1-(7-methoxycarbonylheptyl) bicyclo[4.4.0]deca-2,5,7-triene	C_25_H_40_O_2_	FA methyl ester
41.06	13.22	Ursolic acid methyl ester	C_31_H_50_O_3_	Triterpenoids
42.24	0.54	9,12-Octadecadienoic acid (*Z*,*Z*)-	C_27_H_54_O_4_Si_2_	Unsaturated FA
42.47	0.67	5-Fluoro ADB metabolite 7	C_19_H_26_FN_3_O_3_	Cannabinoid
42.88	3.26	Oleanolic acid	C_30_H_48_O_3_	Triterpenoid
43.94	4.71	Betulin	C_30_H_50_O_2_	Triterpenoid
44.32	2.98	Stigmasterol	C_29_H_48_O	Phytosterol
45.17	11.14	á-Sitosterol	C_29_H_50_O	Phytosterol

*FA = fatty acid.

## 4 Discussion

Traditional pesticides have proven to be effective in reducing pest damage in agricultural areas. However, they come with a number of serious dangers to human health and the environment [41]. Pesticide overuse has the potential to have devastating effects on biodiversity due to the toxicity it may cause in non-target organisms. Residues from pesticides can transfer to groundwater or accumulate in plant tissue, causing gastrointestinal, neurological, and carcinogenic issues in humans [42,43]. As a result, the production and use of biopesticides have increased, gradually replacing some synthetic chemical pesticides. *S. kali* is an annual halophytic plant that grows naturally in environments with high salt concentrations, such as coasts and desert areas. Some species of the genus *Salsola*, including *S. kali*, have a long history of use in traditional Chinese and North African medicine to treat hypertension, inflammation, and gastrointestinal diseases [22,44]. Recent studies have looked at the antimicrobial effects of *Salsola* species extracts on clinically important pathogenic bacterial and fungal strains. However, as far as we know, no studies have been done on the effect of *S. kali* extracts on phytopathogenic microorganisms.

The current study conducted an in vitro experiment, which demonstrated that Gram-negative bacteria, *P. carotovorum, P. atrosepticum*, and *R. solanacearum* exhibited greater sensitivity compared to the control. Differences in sensitivity between the two bacterial groups, Gram-positive and Gram-negative bacteria, have been observed in several studies. Elisha et al. [45] discovered that leaf extracts from nine distinct plant species were more effective against *E. coli*, *Salmonella typhimurium*, and *Pseudomonas aeruginosa* than *S. aureus*, *Enterococcus faecalis*, and *Bacillus cereus*. Makhafola et al. [46] also said that the *Ochna pretoriensis* crude extract had higher average minimum inhibitory concentration (MIC) values against Gram-negative bacteria than against Gram-positive bacteria in their study. However, other previous investigations obtained opposite results [47,48]. This may be attributed to the fact that the efficacy of the plant extract against bacterial pathogens depends on several factors, such as the polarity of the solvent used in the extraction process, the nature of bioactive components, and the variation in cell wall structure between Gram-positive and Gram-negative bacteria [45,49]. Regarding the antifungal study, our findings show that the *S. kali* extract works better against *R. solani* than the other tested *F. oxysporum* fungus. Zhao et al. [50] showed that the *Moutan cortex* extract had a higher inhibitory effect against *R. solani* and *Fusarium* spp. than *Alternaria* spp. and *B. cinerea*. The fungicidal activity of the plant extract depended on the type and concentration of the bioactive compounds, as found by Alotibi et al. [51], who evaluated the antifungal activity of *Rumex vesicarius* L. and *Ziziphus spina-christi* (L.) Desf. aqueous extracts at 1, 10, and 50 mg/mL and found that the increase in concentrations of plant extract had a positive relationship with the growth of fungi such as *F. oxysporum* and *Alternaria* spp. and a negative relationship with *R. solani*. The species-specific differences in the composition of the cell wall and/or membrane might also be responsible for these variations in antifungal efficacy.

There are several secondary metabolites produced by plants that have the potential to act as antibacterial agents. Polyphenols are one of the most abundant secondary metabolites in the class and are classified into four main groups: phenolic acids, flavonoids, stilbenes, and lignans [52,53]. Our research showed the presence of several bioactive phenolic and flavonoid components in the *S. kali* extract using HPLC analysis. Gallic acid was identified as the major phenolic compound in the plant extract, followed by caffeic acid and cinnamic acid. The antimicrobial capability of numerous bioactive substances is typically closely related to the presence of phenolic compounds. Gallic acid has been proven to possess potent bactericidal properties against multi-drug-resistant *E. coli* and effectively hinder the production of bacterial biofilm [54]. Pinho et al. [55] found that gallic acid and caffeic acid from various wild plants can stop the growth of three types of bacteria: *S. epidermidis*, *S. aureus*, and *K. pneumoniae*. Wang et al. [56] demonstrated that the antifungal properties of cinnamic acid against *Sclerotinia sclerotiorum* extend to preventing sclerotia formation as well as mycelial growth. The phenolic acids caused the bacterial cell to explode due to hyperacidification of the plasma membrane and the efflux pump. In the present study, we also observed a significant number of flavonoid compounds in the *S. kali* extract. These bioactive components in the plant extract may be responsible for its antibacterial properties. Flavonoids have the potential to influence protein and RNA production by reducing energy consumption and DNA synthesis. Flavonoids may also inhibit various cellular enzyme synthases involving nucleic acid synthesis, the bacterial respiratory chain, or cell envelope synthesis [57,58].

GC-MS is a proven method for identifying and quantifying bioactive chemicals in plants. The qualitative phytochemical analysis of *S. kali* extract revealed the presence of numerous compounds belonging to different organic classes. Fatty acids, saturated and unsaturated, were the most abundant hydrocarbon compounds identified in this extract. Numerous plant species use fatty acids as a defense mechanism against pathogenic bacteria. Fatty acids’ primary goal is to interfere with the electron transport chain. Furthermore, they decrease nutrition absorption and inhibit enzyme function [59]. The most abundant phytochemical compound found in the GC-MS analysis was the fatty acid n-hexadecanoic acid, which has antioxidant and antifungal properties against *Alternaria alternata* [31,60]. The *n*-hexadecanoic acid exhibited considerable antimicrobial properties against *S. aureus, B. subtilis, E. coli*, and *K. pneumoniae*, with inhibition zones of 7.96, 10.96, 11.10, and 11.93 mm, respectively, at a maximum concentration of 50 µg/mL [61]. The presence of *n*-hexadecanoic acid has been detected in plant extracts of *Benincasa hispida, Carissa congesta, Allium nigrum, Kielmeyera coriacea, Cyrtocarpa procera, Labisia pumila,* and *Rosa indica*. It has been claimed that this compound has antibacterial properties [62]. Furthermore, the in silico technology has been employed to utilize *n*-hexadecanoic acid in the development of phospholipase A (2) inhibitors that are specifically designed. This was achieved by comparing it with other established inhibitors that function as anti-inflammatory medicines [63,64]. The primary target of its activity is the bacterial cell membranes. Furthermore, it impedes cellular energy production, hinders enzyme function, and ultimately leads to bacterial cell destruction. Due to its high level of safety and effectiveness, this antibacterial therapeutic drug has enormous promise [65].

Ursolic acid methyl ester, also known as urs-12-en-28-oic acid or 3-hydroxy-methyl ester, is the second most bioactive compound in our extract. It is a natural triterpenoid compound that has been suggested to have antioxidant properties [66]. Terpenoids have antioxidant properties because they eliminate free radicals. They also have antibacterial or bactericidal properties because they can inhibit oxygen uptake and oxidative phosphorylation [67]. In a study by dos Santos et al. [68], they looked at the antibacterial activity of ursolic acid or its combination with other tripenoid such as oleanolic acid. They found that the mix of ursolic and oleanolic acids (1 + 2) was the most effective against *Bacteroides fragilis*, with an MIC value of 20 μg/mL. The combination had higher activity than ursolic acid alone (MIC = 80 μg/mL), suggesting a potential synergistic effect. The 1 + 2 combination was potent against *Actinomyces naeslundii* and *Porphyromonas gingivalis*, with MIC values of 20 and 40 μg/mL, respectively. Andrade et al. [69] found that ursolic acid was effective against most bacteria tested, with MIC values of 20 μg/mL for *A. naeslundii* and *P. gingivalis*, 80 μg/mL for *B. fragilis*, and 90 μg/mL for *Prevotella nigrescens*. Oleanolic acid successfully inhibited *A. naeslundii* and *P. gingivalis* strains, with MIC values of 20 and 40 μg/mL, respectively [70].

Mallavadhani et al. [71] have demonstrated that lipophilicity plays a crucial role in the development of biological agents. The scientists stated that molecules with carbon chains exceeding C-10 are highly lipophilic and so well-suited for pharmacological testing. Based on these discoveries, two lipophilic derivatives of ursolic acid with 3-*O*-fatty acid ester chains (1b and 1c) were synthesized [68]. The 3β-dodecanoate (1b) and 3β-octadecanoate (1c) derivatives of ursolic acid did not result in decreased MIC values against the tested bacteria, while the C-28 methyl ester derivative (1d) improved the activity against *B. fragilis* only, with an MIC value of 20 μg/mL. The discrepancies in results could be attributed to the varied bacteria species used in the experiments and to the processes involved in the action. 9,12-Octadecadienoic acid was the third most common compound in our ethanolic extract. It was found in large amounts in many parts of plants, like the *n*-hexane extract of *B. hispida* and *Cucurbita moschata* seeds, which had two main components in large amounts. The most abundant ingredient was 9, 12-octadecadienoic acid (ZZ) with a concentration of 79.88 and 59.71%, respectively. The second major molecule was *n*-hexadecanoic acid, which accounted for 16.95 and 37.38% of the two plant extracts, respectively [72]. Krishnaveni et al. [73] reported that *Gossypium* seed powder GCMS contained 9,12-octadecadienoic acid as an abundant phytochemical compound, and the authors evaluated the ethanolic extract of the *Gossypium* seed powder against fungal and bacterial strains and found that *Gossypium* seed powder has fungicidal properties against *Aspergillus niger* and *Aspergillus flavus*. In addition, it exhibits activity against *Escherichia coli* and *S. aureus*. The seed powder exhibits antifungal and antibacterial properties at concentrations of 50 and 100 μg. It was discovered to have a higher level of bactericidal activity. The suggested antibacterial action seen in *Gossypium* seed powder is attributed to the presence of phytochemicals in it. Additionally, á-sitosterol and stigmasterol, phytosterol compounds that are present in high concentrations in the studied plant extract, have been shown to exhibit antimicrobial effects. Prior research has shown that stigmasterol and β-sitosterol, which were extracted from the stems of *Neocarya macrophylla* and *Punica granatum*, possess antibacterial properties [74,75]. Because of their similarity to the sterols naturally present in bacterial cells, phytosterols can be used to kill bacteria by replacing the regular sterols in the bacterial cell membrane. Disruption of the cell membrane is one of the potential processes that might explain the activity of sterols on microorganisms [76].

## 5 Conclusion

In conclusion, the ethanolic extract from the *S. kali* aerial parts effectively fought against the tested bacterial and fungal strains, as well as inhibiting their growth. Because of its high phenolic and flavonoid content, the extract had a significant inhibitory effect on *R. solani*, as well as other bacterial strains such as *P. carotovorum*, *P. atrosepticum*, and *Ralstonia solanacearum*. The findings highlight the value of *S. kali* extract as a bioactive chemical reservoir for agricultural applications, particularly in plant disease management and crop protection.
